# Aortic stiffness as a marker of cardiac function and myocardial strain in patients undergoing aortic valve replacement

**DOI:** 10.1186/1749-8090-9-102

**Published:** 2014-06-17

**Authors:** Emaddin Kidher, Leanne Harling, Hutan Ashrafian, Hatam Naase, Darrel P Francis, Paul Evans, Thanos Athanasiou

**Affiliations:** 1 The Department of Surgery and Cancer, Imperial College London, St Mary’s Hospital, 10th Floor, QEQM Wing, St Mary’s Campus, London W2 1NY, UK; 2 Imperial College Healthcare NHS Trust, Hammersmith Hospital, London, UK; 3 International Centre for Circulatory Health, Imperial College Healthcare NHS Trust, St Mary’s Hospital, London, UK; 4 Department of Cardiovascular Science, University of Sheffield, Sheffield, UK

**Keywords:** Pulse wave velocity, Aortic stiffness, PWV, Myocardial strain, B-type natriuretic peptide, BNP, Aortic valve replacement, AVR

## Abstract

**Background:**

Cardiac function and myocardial strain are affected by cardiac afterload, which is in part due to the stiffness of the aortic wall. In this study, we hypothesize that aortic pulse wave velocity (PWV) as a marker of aortic stiffness correlates with conventional clinical and biochemical markers of cardiac function and perioperative myocardial strain in aortic valve replacement (AVR).

**Methods:**

Patients undergoing AVR for aortic stenosis between June 2010 and August 2012 were recruited for inclusion in this study. PWV, NYHA class and left ventricular (LV) function were assessed pre-operatively. PWV was analysed both as a continuous and dichotomous variable according to age-standardized reference values. B-type natriuretic peptide (BNP) was measured pre-operatively, and at 3 h and 18-24 h after cardiopulmonary bypass (CPB). NYHA class, leg edema, and LV function were recorded at follow-up (409 ± 159 days).

**Results:**

Fifty-six patients (16 females) with a mean age of 71 ± 8.4 years were included, with 50 (89%) patients completing follow-up. The NYHA class of PWV-norm patients was significantly lower than PWV-high patients both pre- and post-operatively. Multiple logistic regression also highlighted PWV-cut off as an independent predictor of NYHA class pre- and post-operatively (OR 8.3, 95%CI [2.27,33.33] and OR 14.44, 95%CI [1.49,139.31] respectively). No significant relationship was observed between PWV and either LV function or plasma BNP.

**Conclusion:**

In patients undergoing AVR for aortic stenosis, PWV is independently related to pre- and post-operative NYHA class but not to LV function or BNP. These findings provisionally support the use of perioperative PWV as a non-invasive marker of clinical functional status, which when used in conjunction with biomarkers of myocardial strain such as BNP, may provide a holistic functional assessment of patients undergoing aortic valve surgery. However, in order for PWV assessment to be translated into clinical practice and utilised as more than simply a research tool, further validation is required in the form of larger prospective studies specifically designed to assess the relationship between PWV and these functional clinical outcomes.

## Background

In a normal, healthy, elastic aorta, radial expansion during systole acts as a volume and pressure reservoir (“cushioning effect”). This is possible because the aorta, particularly proximally, is richer in elastin than the rest of the arterial tree [1-3]. This property is of particular importance for coronary perfusion after aortic valve closure. When the elastic properties diminish, the aorta becomes stiffer, and the change in aortic diameter during systole and diastole is reduced. The rigidity of the aorta increases pulse wave speed, causing the reflected pressure wave towards the heart to occur at the beginning of systole, rather than during diastole. Consequently, increased systolic blood pressure and elevated cardiac afterload results in left ventricular (LV) hypertrophy. Furthermore, the absence of diastolic augmentation reduces coronary flow during diastole and widens the pulse pressure (PP) [1,4].

Aortic stiffness increases with age [3,5], and aortic PWV is specifically associated with an increased risk of cardiovascular events in the general population [6]. Meta-analysis of 17 longitudinal studies including 15,877 participants revealed that an increase of aortic PWV of 1 m/s increases total CV events, CV mortality, and all-cause mortality by 14%, 15%, and 15%, respectively [7]. In 2010, the Reference Values for Arterial Stiffness’ Collaboration published reference and normal values for PWV [8]. “Normal” values were derived from a population with no cardiovascular risk factors (other than age and sex), while “reference” values were derived from a population with various risk factors, according to age and blood pressure. The availability of such normal and reference values for PWV per age group makes identifying individuals with abnormal PWV possible and practical without the need for recruiting matched control groups as long as the methodology of measuring aortic PWV remains the same. Among many indices of measuring aortic stiffness, PWV is the gold standard parameter of arterial stiffness as it is the most accepted, valid, reproducible, and widely used [1,2,8]. As such, it is the method recommended by the European Network for Non-invasive Investigation of Large Arteries for direct measure of arterial stiffness [2].

Cardiac function assessment includes appraisal of clinical, echocardiography and biochemical parameters. The use of cardiac or myocardial biomarkers is essential for assessing and monitoring cardiovascular diseases and for predicting outcomes. Biomarkers of myocardial damage, such as troponins, are different from biomarkers of myocardial strain, such as natriuretic peptides. Natriuretic peptides (NPs) are released from the heart in response to pressure and volume overload to induce vasodilation, natriuresis, and diuresis. In AS populations, NPs are related to the severity and/or functional status symptoms of AS disease, and increase progressively with worsening NYHA functional class [9]. In patients who undergo valve replacement, NP levels may potentially predict survival and post-operative LV function [10,11].

Pulse wave velocity (PWV) is known to be significantly associated with plasma BNP level in both healthy [12] and hypertensive patients [13]. Indeed, in their study of coronary artery disease patients, Sakuragi et al. propose that increased PWV may in fact directly result in a rise in BNP [14]. Aortic PWV has also been found to be independently associated with LV ejection fraction (EF) in patients with type 1 diabetes mellitus [15], and to be inversely correlated with LV function in coronary artery disease [16]. However, despite these findings, no study currently addresses the relationship between aortic stiffness and BNP in aortic valve replacement (AVR) patients, and the association between aortic stiffness, cardiac function and clinical outcomes after aortic valve surgery remains to be assessed.

We hypothesize that aortic stiffness is related to cardiac function and perioperative myocardial strain in patients undergoing aortic valve replacement. As such, this study aims to assess the relationship between aortic PWV and: (a) pre- and post- operative cardiac function assessed by NHYA class and echocardiography; (b) pre- and post- operative BNP as a marker of myocardial strain; (c) the need for post-operative inotropes; and (d) the development of post-operative arrhythmia.

## Methods

### Overview

Ethical approval (11/H0709/3, North London Research Ethic Committee 3) and participant informed consent were obtained prior to undertaking this study. Patients undergoing AVR for moderate to severe AS were eligible. Patients were excluded if they had a history of or presented with: (i) aortic dissection; (ii) thoracic aortic (more than just the root) or abdominal aortic aneurysm; (iii) Marfan’s syndrome; or (iv) chronic kidney disease. All patient recruitment and data collection was performed prospectively.

Aortic PWV, NYHA class, LV function, and plasma BNP were assessed pre-operatively. BNP level was re-measured 3 h post-CPB and on day one (18–24 h post-CPB) after AVR. Post-operative inotrope requirement for more than 6 hours and any cardiac arrhythmia were also recorded. NYHA class, LV function, and the presence of leg edema were recorded at the study-specific follow-up visit (409 ± 159 days) post-operatively.

PWV was analyzed as a continuous variable using traditional methods. In addition, PWV was analyzed as a dichotomous variable (PWV-norm group vs. PWV-high group) by categorizing patients according to the published European normal values of PWV for the matched age group (subtracted method) [8]. As such, patients were divided into two groups:

A- PWV-norm group: PWV equal or below normal reference values for their age group.

B- PWV-high group: PWV above the normal reference values for their age group.

### Pulse wave velocity measurement protocol

Prior to measurement all participants abstained from tobacco, alcohol, tea or coffee for at least 2 hours. Patients were rested for 10 minutes in a quiet, temperature-controlled room (22-25°C) before the hemodynamic measurements were taken. Brachial blood pressure (BP) and heart rate were measured using a digital sphygmomanometer (Criticare, Model 506 N3, Waukesha, USA). BP measurements were taken before and after PWV assessment and the mean used for analysis. Aortic PWV (carotid-femoral PWV) was measured with an automatic applanation tonometry system (SphygmoCor Vx, AtCor Medical, Australia). Briefly, with the patient resting in a supine position, ECG-gated pulse waveforms were obtained sequentially from the common carotid and then femoral arteries. Propagation time of the pulse wave was measured from the foot of the carotid waveform to that of the femoral waveform referenced to the R wave on the recorded ECG. The transit distance (mm) was measured over the body surface by subtracting the supra-sternal notch to carotid site distance from the supra-sternal notch to femoral site distance. PWV was subsequently calculated in m/s using the Sphygmocor device by dividing the travelled distance by the propagation time. Three to five readings were obtained per patient and PWV was determined by averaging the measurements meeting quality control parameters outlined by SphygmoCor.

### Clinical assessment

Clinical history and examination including NYHA functional classification and the presence of leg oedema were recorded both pre-operatively and at follow-up.

### Echocardiography assessment

Cardiac function was determined according to the LV functional class derived from pre-and post-operative echocardiography. LV function was classified into good (LV EF >50%), moderate (LV EF 31%–50%), poor (LV EF 21%–30%), and very poor (LV EF equal or ≤20%) [17].

### Biochemical assessment of B-type natriuretic peptide (BNP)

Myocardial strain was assessed by measuring BNP pre-operatively, 3 h post-CPB, and on day one (18–24 h post-CPB) after AVR. Venous blood samples were collected in Ethylenediaminetetraacetic acid (EDTA)-containing tubes and immediately centrifuged to separate plasma. BNP was measured using a chemiluminescent immunoassay (Biosite Triage, Biosite Incorporated, San Diego, CA, USA, currently called Alere Triage MeterPro, Alere Ltd, Stockport, UK), for which the accuracy, analytic sensitivity, and stability characteristics have been previously validated and described [18,19]. All BNP measurements were performed within 30 minutes of sample collection by a single operator (EK).

### Statistical analysis

Statistical analysis was conducted using IBM SPSS statistics 20.0 (IBM Corp., Armonk, NY, USA). Patient characteristics and results are expressed as means ± standard deviation for continuous variables and as frequencies for categorical variables. *P*-values < 0.05 were considered statistically significant. Test for normality was carried out on all variables, and non-normally distributed variables transformed for the purpose of regression analysis. PWV was analyzed both as a continuous and dichotomous variable (PWV cut-off). Inter-group comparison was carried out using an independent samples *t*-test or non-parametric equivalent (Mann–Whitney U test) for continuous variables, and Pearson Chi-square or Fisher’s exact test for categorical variables. The association between any two variables was tested with bivariate correlation analysis using Spearman's rank-order correlation (both continuous), point biserial correlation (continuous vs. dichotomous), Phi test (both dichotomous variables), or Carmer V (both nominal other than 2 × 2). All variables with significant correlation, in addition to age and gender, were then included in two models of multiple regression, linear or logistic based on the dependent variable (enter method): (1) Model 1: including PWV as a continuous predictor; and (2) Model 2: including PWV cut-off as a dichotomous predictor.

## Results

### Descriptive results

Between June 2010 and August 2012, 56 patients (16 females) with a mean (±SD) age of 71 ± 8.4 years were included in this study. Fifty (89%) patients attended follow-up, however the remaining 6 refused to return after their initial enrollment at the pre-operative stage. No mortality or cerebrovascular events were recorded during the follow-up period. Thirty-five (62.5%) patients had PWV measurements equal or below the reference value for their age group (PWV-norm group), and 21 patients (37.5%) had PWV measurements above the reference value for their age group (PWV-high group). There was no significant difference between the PWV-norm and PWV-high groups in terms of age, gender, severity of pre-operative aortic stenosis, classical hemodynamic measurements or other clinical characteristics (Table 1).

**Table 1:** Demographic and clinical characteristics

**Parameter**	**Total**	**PWV-norm**	**PWV-high**	***P*-value**
	**n = 56**	**n = 35**	**n = 21**	
Male	40 (71.4%)	27 (77.1%)	13 (61.9%)	0.36
Age (years)	71 ± 8.4	70.2 ± 9	72 ± 8	0.43
Age range (years)	53 - 90	53 - 90	56 - 85	
White Caucasian [n (%)]	55 (98%)	35 (100%)	20 (95%)	0.37
BMI (kg/m^2^)	27.2 (4.2)	26.5 (4.3)	28.1 (4.1)	0.18
SBP (mmHg)	136 ± 24	132 ± 27	142 ± 18	0.14
DBP (mmHg)	76 ± 11	75 ± 12	77 ± 11	0.58
MAP (mmHg)	97 ± 12	96 ± 13	98 ± 11	0.56
DM [n (%)]	8 (14.3%)	4 (11.4%)	4 (19.1%)	0.43
Smoking [n (%)]	2 (3.6%)	2 (5.7%)	0 (0%)	0.21
Cholesterol (mmol/l)	4.5 ± 1.2	4.6 ± 1.3	4.4 ± 0.8	0.60
Hypertension [n (%)]	38 (67.9%)	22 (62.9%)	16 (76.2%)	0.38
PVD [n (%)]	2 (3.6%)	0 (0%)	2 (9.5%)	0.13
EuroSCORE (Logistic)	5.5 ± 4.3	5.1 ± 4	6.1 ± 4.7	0.23
Concomitant CABG	15 (26.8%)	10 (28.5%)	5 (23.8%)	0.76
CPB time (minutes)	87 ± 23	87 ± 20	87 ± 27	0.95
Hospital stay (days)	6.7 ± 1.7	6.6 ± 1.6	6.7 ± 1.8	0.84
PWV (m/s)	9.3 ± 2.2	8.1 ± 1.5	11.3 ± 1.6	**<0.001**

Values are shown as n (%) for categorical variables and the mean ± standard deviation for continuous variables. bold values indicate statistically significant values. *Abbreviations:DM* diabetes mellitus, *BMI* body mass index, *SBP* systolic blood pressure, *DBP* diastolic blood pressure, *PP* pulse pressure, *MAP*, mean arterial blood pressure, *PVD* peripheral vascular disease, *CABG* coronary artery bypass graft surgery, *CPB* cardiopulmonary bypass, *PWV* pulse wave velocity.

### Pulse wave velocity and NYHA functional classification

There was a significant difference in NYHA class between the two groups in favor of PWV-norm pre- and post-operatively (Table 2). Pre-operatively, 0% of the PWV-high group was categorized as NYHA class I compared to 46% of the PWV-norm group, while the percentage of patients in NYHA classes II & III was much higher in the PWV-high group. Post-operatively there was an overall improvement in NYHA status in both groups, with no patients categorized as NYHA class III after surgery. The number of patients classified as NYHA class II fell from 43% to 3% in the PWV-norm group and from 71% to 45% in the PWV-high group (Table 2).

**Table 2:** Cardiac assessment characteristics

**Parameter**	**Total**	**PWV-norm**	**PWV-high**	***P*-value**
	**n = 56**	**n = 35**	**n = 21**	
**Pre-operative**				
Aortic valve area (cm^2^)	0.73 ± 0.2	0.74 ± 0.2	0.71 ± 0.2	0.67
AVMG (mmHg)	48 ± 13	50 ± 13	46 ± 11	0.27
AVPG (mmHg)	82 ± 24	86 ± 27	76 ± 19	0.13
Ejection fraction	59 ± 15	58 ± 15	61 ± 16	0.56
LVDDiam (cm)	4.79 ± 0.72	4.78 ± 0.75	4.82 ± 0.69	0.85
LVSDiam (cm)	3.34 ± 0.83	3.25 ± 0.76	3.47 ± 0.94	0.37
NYHA (n [%])				**<0.01**
Class I	16 (29%)	16 (46%)	0 (0%)	
Class II	30 (53%)	15 (43%)	15 (71%)	
Class III	10 (18%)	4 (11%)	6 (29%)	
LV function (n [%])				0.97
Good	41 (76%)	25 (76%)	16 (76%)	
Moderate	10 (18%)	6 (18%)	4 (19%)	
Poor	3 (6%)	3 (6%)	3 (5%)	
BNP pg/ml	134 ± 183	88 ± 88	214 ± 264	0.09
**Immediate post-operative**				
BNP pg/ml (3 h post-CPB)	112 ± 143	85 ± 81	158 ± 199	0.32
BNP pg/ml (18 h post-CPB)	334 ± 179	288 ± 157	406 ± 191	**0.02**
Inotropes (n [%])	16 (29%)	8 (23%)	8 (40%)	0.22
Arrhythmia (n [%])	23 (41%)	16 (46%)	7 (33%)	0.41
**Post-operative (409 ± 159 days)**	n = 50			
NYHA (n [%])				**<0.001**
Class I	40 (80%)	29 (97%)	11 (55%)	
Class II	10 (20%)	1 (3%)	9 (45%)	
LV function (n [%])				1.0
Good	40 (87%)	23 (85%)	17 (89%)	
Moderate	6 (13%)	4 (15%)	2 (10%)	
Leg edema (n [%])	7 (14%)	1 (3%)	6 (30%)	**0.01**

Values are shown as n (%) for categorical variables and means ± standard deviation for continuous variables. bold values indicate statistically significant values. *Abbreviations: AVMG* aortic valve mean gradient, *AVPG* aortic valve peak gradient, *LVDDiam* left ventricular diastolic diameter, *LVSDiam* left ventricular systolic diameter, *NYHA* New York Heart Association, *LV* left ventricle, *BNP* B-type natriuretic peptide.

Bivariate correlation demonstrated a significant association between NYHA class and PWV, particularly where PWV was measured as a dichotomous variable (Table 3). Chi-square analysis was used to identify any association between PWV groups (PWV cut-off) and NYHA class. There was a statistically significant association between PWV cut-off and NYHA pre- (*X*^*2*^ = 13.76, *P* < 0.01) and post- operatively (*X*^*2*^ = 13.02, *P* < 0.001). Multiple logistic regression adjusted for age, gender, and other significantly correlated variables (Table 4), confirmed these findings and that PWV (continuous variable) was an independent predictor of pre-operative NYHA class (OR 1.43, 95% CI [1.08–1.91], but not of post-operative NYHA. PWV cut-off (dichotomous) was a stronger predictor of NYHA class than PWV-norm (continuous). Patients in the PWV-high group were 8.3 times more likely to be in a higher NYHA class pre-operatively and 14.4 times more likely to be in a higher NYHA class post-operatively than PWV-norm patients (Table 4).

**Table 3:** Bivariate correlation between potential predictors, cardiac function and myocardial strain parameters

	**Age**	**Gender (male)**	**DM**	**Hypertension**	**MAP**	**AVMG (pre–op)**	**CPB time**	**PWV value**	**PWV cut–off**
**Pre–operative**									
LV function	0.18	-0.19	-0.11	-0.38^**^	-0.26	-0.13	-0.04	-0.12	-0.03
NYHA class	0.09	-0.05	0.18	-0.11	0.04	-0.14	-0.08	0.36^**^	0.50^**^
BNP	0.27	-0.35^*^	-0.19	-0.07	-0.30^*^	0.10	-0.00	0.21	0.34^*^
**Immediate post-operative**									
BNP (3 h post-CPB)	0.22	-0.34^*^	-0.23	-0.13	-0.20	-0.14	-0.15	0.09	0.26
BNP (18 h post-CPB)	0.24	-0.26	-0.29^*^	-0.01	0.03	-0.09	-0.04	0.24	0.32^*^
Inotropes	0.24	-0.24	-0.11	-0.15	-0.06	-0.08	-0.14	0.35^*^	0.18
Arrhythmia	0.24	0.13	-0.14	0.11	-0.20	0.04	-0.07	0.06	-0.12
**Post–operative**									
LV function	0.07	0.10	0.23	-0.12	-0.11	-0.1	-0.08	-0.14	-0.06
NYHA class	0.03	-0.24	0.28^*^	0.15	-0.12	-0.15	0.15	0.20	0.51^**^
Leg edema	-0.02	-0.13	0.02	0.05	0.08	-0.21	0.03	0.07	0.38^**^

Values are shown as correlation coefficients (***r***); bold values indicate statistically significant values; ^*^Correlation is significant at the 0.05 level (2-tailed); ^**^Correlation is significant at the 0.01 level (2-tailed). *Abbreviations: DM* diabetes mellitus, *MAP* mean arterial blood pressure, *AVMG* aortic valve mean gradient, *CPB* cardiopulmonary bypass, *PWV* pulse wave velocity, *LV* left ventricle, *NYHA* New York Heart Association Functional Classification, *BNP* B-type Natriuretic peptides.

**Table 4:** Multiple logistic regression (binary and ordinal) to identify predictors of cardiac function and myocardial strain

**Predictors**	**Pre-operative**		**Post-operative**				
	**OR (95% CI)**		**OR (95% CI)**				
	**LV function**	**NYHA class**	**Inotropes**	**Arrhythmia**	**LV function**	**NYHA class**	**Leg edema**
**Model 1**							
PWV value	0.73	1.43^*^	1.33	0.94	0.76	1.20	1.09
	(0.52–1.05)	**(1.08–1.91)**	(0.94–1.88)	(0.71–1.26)	(0.46–1.23)	(0.76–1.91)	(0.71–1.67)
Age	1.11^*^	0.97	1.03	1.07	1.05	0.93	0.97
	**(1.01–1.23)**	(0.91–1.04)	(0.94–1.12)	(0.99–1.16)	(0.93–1.19)	(0.81–1.07)	(0.86–1.09)
Gender (male)	0.35	1.11	0.36	1.80	1.80	0.34	0.47
	(0.08–1.49)	(0.35–3.57)	(0.09–1.45)	(0.49–6.56)	(0.18–18.01)	(0.04–2.36)	(0.08–2.66)
DM						5.01	
						(0.72–34.51)	
Hypertension	0.20^*^						
	**(0.05–0.81)**						
**Model 2**							
PWV cut-off	0.91	8.3^**^	1.78	0.55	0.81	14.44^*^	11.77^*^
(PWV-high)	(0.21–3.84)	**(2.27–33.33)**	(0.49–6.43)	(0.16–1.84)	(0.12–5.41)	**(1.49–139.31)**	**(1.26–109.77)**
Age	1.06	1.02	1.06	1.07^*^	1.03	0.97	0.97
	(0.98–1.15)	(0.95–1.08)	(098–1.15)	**(1.0–1.15)**	(0.91–1.15)	(0.84–1.10)	(0.86–1.09)
Gender (male)	0.47	1.21	0.34	1.65	2.10	0.43	0.62
	(0.11–1.92)	(0.38–4.0)	(0.08–1.30)	(0.45–6.03)	(0.19–22.13)	(0.05–3.52)	(0.09–4.18)
DM						4.45	
						(0.47–41.66)	
Hypertension	0.18^*^						
	**(0.04–0.71)**						

Values are shown as odd ratio (95% CI), bold values indicate statistically significant values; ^*^is significant at the 0.05 level (2-tailed); ^**^is significant at the 0.01 level (2-tailed); Model 1 includes PWV value as a continuous predictor; Model 2 includes PWV cut-off as a dichotomous predictor. *Abbreviations: LV* left ventricle, *NYHA* New York Heart Association (NYHA) Functional Classification, *PWV* pulse wave velocity, *DM* diabetes mellitus.

### Pulse wave velocity and left ventricular (LV) function

There was no significant difference in categorical groupings of LV function (good, moderate, and poor) between the two PWV groups either pre- or post-operatively (Table 2). Although the overall improvement in LV function post-operatively did not reach statistical significance (*P* = 0.058), no patients were categorized as ‘poor’ LV function post-operatively. Further correlation and multiple logistic regression (Tables 3 and 4) analyses confirm the absence of any relationship between PWV and LV function, whilst also demonstrating a significant correlation between pre-operative LV function and both age and hypertension (OR 1.11, 95% CI [1.01–1.23] and 0.20 [0.05–0.81] respectively).

### Pulse wave velocity and myocardial strain

Plasma BNP (pg/ml) was similar between PWV-norm and PWV-high groups pre-operatively and at 3 h post-CPB, however it was significantly higher in the PWV-high group at 18 h after CPB (288 ± 157 vs. 406 ± 191, *P* =0.02; Table 2). PWV as a dichotomous variable (PWV-cut-off) was significantly associated with both pre-operative BNP (*r* = 0.34) and BNP at 18 h post-CPB (*r* = 0.32). However, this was not seen when PWV was analysed as a continuous variable. Multiple regression analysis (Table 5) demonstrates that age and mean atrial pressure (MAP) were independently related to BNP pre-operatively, and gender independently related to BNP at 3 h post-CPB. PWV cut-off and diabetes were the only two variables independently related to BNP at 18 h after CPB.

**Table 5:** Multiple linear regression to identify predictors of the myocardial strain biomarker BNP

**Predictors**	**BNP (pre-op.)**^**A**^	**BNP (3 h post-CPB)**^**A**^	**BNP (18 h post-CPB)**^**A**^
**Model 1**			
PWV value	0.06	0.11	0.18
	(0.72)	(0.51)	(0.29)
Age	0.26	0.29	0.01
	(0.11)	(0.09)	(0.95)
Gender (male)	-0.20	**-0.38**	-0.12
	(0.15)	**(0.0.1)**	(0.43)
MAP	**-0.36**		
	**(0.01)**		
DM			**-0.30**
			**(0.05)**
**Model 2**			
PWV cut-off	0.20	0.09	**0.32**
(PWV-high)	(0.14)	(0.51)	**(0.03)**
Age	**0.27**	0.21	0.06
	**(0.05)**	(0.14)	(0.65)
Gender (male)	-0.17	**-0.34**	-0.09
	(0.22)	**(0.02)**	(0.51)
MAP	**-0.35**		
	**(0.01)**		
DM			**-0.33**
			**(0.03)**

Values are shown as beta (*P*-value), bold values indicate statistical significance; ^A^Log transformation. Model 1 includes PWV value as a continuous predictor; Model 2 includes PWV cut-off as a dichotomous predictor. *Abbreviations: DM* diabetes mellitus, *MAP* mean arterial blood pressure, *PWV* pulse wave velocity, *BNP* B-type natriuretic peptides.

### Clinical findings

Leg edema was present in 14% (n = 7) cases at follow-up, 85% of which were in the PWV-high group (*P* = 0.01). Bivariate and multiple logistic regression (Tables 3 and 4) revealed a significant relationship between PWV cut-off and post-operative leg edema (*r* = 0.38, OR 11.77, 95% CI [1.26–109.77]). Patients in the PWV-high group required more post-operative inotropes (>6 hours) (40% vs. 23%), and developed less arrhythmia than the PWV-norm group (33% vs. 46%) although neither outcome reached statistical significance (Table 2). PWV as a continuous variable significantly correlated with the need for inotropes beyond 6 hours post-operatively (*r* = 0.35; Table 3), however, this association disappeared in multiple logistic regression (OR 1.33 95%CI [0.94–1.88]; Table 4).

## Discussion

This is the first study of its kind to address the relationship between aortic PWV and NYHA class in surgical patients. The predictive value of NYHA for survival, quality of life, and other outcomes has been reported extensively [20-22]. However, NYHA may be limited by the poor correlation of patient reports with cardiopulmonary exercise testing, and significant inconsistencies have been noted between classifications made by different observers (physicians), particularly in classes II and III [23]. As such, a non-invasive quantitative measure that correlates strongly with NYHA classification is of great clinical interest, with the potential to provide a more robust and reproducible predictor of morbidity, mortality and quality of life outcomes.

The results of this study demonstrate a significant improvement in NYHA functional class following AVR, supporting the findings of previous studies [24]. A significant correlation between NYHA class and PWV was also observed in AS patients both pre- and post- AVR, again consistent with findings of other studies examining this relationship in heart failure and ischaemic heart disease [25,26]. In addition, when examining clinical markers of cardiac function, PWV cut-off predicted the likelihood of developing post-operative leg edema despite good LV function.

Controversially, we observed no significant relationship between PWV and LV function as assessed by echocardiography. It is possible that this finding reflects the difference between LV ejection fraction as a specific measure of cardiac function and less-specific clinical assessments such as NYHA class, which may be influenced by non-cardiac factors. However, it is also possible that although LV function is commonly employed in clinical studies, its use to reflect global cardiac function may be inappropriate as it fails to distinguish patients with predominantly diastolic heart failure in whom LV function may be normal [27]. Regrettably, we did not assess diastolic dysfunction parameters in our study, which if included may have aligned the relationship between PWV and echocardiographic assessment with that of clinical assessment (NYHA). In addition, as over 75% of our patients were categorized as “good” LV function both pre- and post-operatively, the low numbers of patients with impaired LV function may have led to our study being underpowered to establish any significance between PWV and this outcome.

Consistent with the absence of correlation between PWV and LV function in our sample, we did not identify any significant association between PWV as a continuous variable and BNP as a marker of myocardial strain. This finding appears to be contradictory to the positive correlation that has been observed in an otherwise healthy population and in patients with both diabetes and coronary artery disease [12-14]. However, when examining PWV as a dichotomous variable (PWV-cut-off) a positive correlation similar to that previously reported is observed both pre-operatively and at 18 hours post-CPB. It is therefore possible that due to its small sample size, our study is underpowered to detect significant correlation when PWV is expressed as a continuous variable. Alternatively however, the pathology of aortic valve stenosis may confound and attenuate the effect of aortic stiffness on LV function and BNP release. As such, this finding requires ongoing research with increased sample size to definitively answer this question.

### Limitations

The results presented here should be considered in the context of a number of limitations. Firstly, as previously mentioned, the use of LV ejection fraction as a marker of cardiac function may have led to the inappropriate classification of patients with diastolic dysfunction in the ‘good’ cardiac function group. Second, the relatively small sample size of our study may have left it underpowered to detect significance for relationships such as that between PWV and LV function. Third, despite the significant relationship between PWV cut-off and post-operative leg oedema (r = 0.38, OR 11.77, 95% CI [1.26–109.77]), leg oedema is a subjective measure with the potential to be influenced by several potential confounders arising during the perioperative period. Furthermore, the exact mechanism of this relationship is unknown, and further research is therefore required to evaluate this association in more detail.

Finally, we did not evaluate other biological factors such as tumor necrosis factor alpha, high sensitivity C-reactive protein, plasminogen activator inhibitor-1, and elastin-derived peptide, which may have provided an explanation for the observed difference in PWV in the otherwise demographically similar population studied here. In addition, future larger studies should also examine the role of potential confounders not assessed here that may affect the relationship between PWV, cardiac function and myocardial strain. These include the degree of aortic atherosclerosis, aortic calcification, type and duration of pharmacotherapy.

## Conclusions

Aortic stiffness measured by aortic PWV is independently associated with pre- and post-operative NYHA and leg edema after aortic valve replacement, but not to LV function or BNP. These findings provisionally support the use of perioperative PWV as a non-invasive marker of clinical functional status, which when used in conjunction with biomarkers of myocardial strain such as BNP, may provide a holistic functional assessment of patients undergoing aortic valve surgery. However, in order for PWV assessment to be translated into clinical practice and utilised as more than simply a research tool, further validation is required in the form of larger prospective studies specifically designed to assess the relationship between PWV and these functional clinical outcomes.

The preliminary data provided in this study may now be used to develop larger multicenter projects evaluating the role of arterial stiffness and neurohormonal predictors in the assessment of cardiac surgical patients. Furthermore, as a quantitative but non-invasive marker of functional status, PWV may help to improve the power of operative risk prediction models thus optimizing perioperative assessment and subsequently improving surgical outcomes.

## Competing interests

The authors declare that there is no competing interests. Alere Ltd., Stockport, UK provided the point-of-care Alere Triage MeterPro and tests strips for BNP free of charge, but they had no control or influence on the study design, data analysis or publications.

## Authors’ contributions

TA provided the original idea for this study and supervised this research. Patients were recruited and data collected by EK. EK and HN processed the biological samples supervised by PE. Data analysis was performed by EK and TA. EK, LH and HA drafted and finalized the manuscript. Cardiology support and supervision was provided by DF. All authors read and agreed the final manuscript prior to submission.
